# Electron Beam Irradiation for Impact Strength Enhancement of Kevlar Fiber-Reinforced Polypropylene

**DOI:** 10.3390/polym18101231

**Published:** 2026-05-18

**Authors:** Hideki Kimura, Yusuke Kobayashi, Hirotaka Irie, Kouhei Sagawa, Helmut Takahiro Uchida, Michael C. Faudree, Michelle Salvia, Yoshitake Nishi

**Affiliations:** 1 Graduate School of Engineering, Tokai University, 4-1, Kitakaname, Hiratsuka 259-1292, Japan; kimura@tokai.ac.jp (H.K.); yk@tokai.ac.jp (Y.K.); suiso001@icloud.com (H.I.); helmutuchida@tokai.ac.jp (H.T.U.); west@tokai.ac.jp (Y.N.); 2 Graduate School of Science & Technology, Tokai University, 4-1, Kitakaname, Hiratsuka 259-1292, Japan; 3 Faculty of Liberal Arts and Science, TCU (Tokyo City University), Yokohama 224-8551, Japan; 4 Laboratoire de Tribologie et Dynamique des Systemes (LTDS), ECL (Ecole Centrale de Lyon), CEDEX, 69134 Ecully, France; michelle.salvia@ec-lyon.fr; 5 KISTEC (Kanagawa Institute of Industrial Science & Technology), 705-1, Shimoimaizumi, Ebina 243-0435, Japan

**Keywords:** composite, thermoplastic, polypropylene, aramid fiber, interlayered, electron beam, impact strength

## Abstract

Presently, there is little to no literature that investigates the effect of electron beams on para-aramid (Kevlar^®^) fiber polymer (KFRP) composites. Therefore, we assessed the effect of homogeneous low-potential electron beam irradiation (HLEBI) on Kevlar-reinforced recyclable thermoplastic (TP) polypropylene (PP) (KFRPP). Samples were assembled in an interlayered configuration of four-sized KF plies between five PP sheets [PP1-KF1-PP2-KF2-PP3-KF2-PP2-KF1-PP1] designated [PP]_5_[KF]_4_, which were hot-pressed at 493 K at 4 MPa for 7 min. Experimental results show when an HLEBI setting of 250 kV cathode potential (*V*_c_) at an 86 kGy dose is applied to finished sample surfaces, the Charpy impact strength (*a*_uc_) at median fracture probability (*P*_f_ of 0.50) is increased 59% from 72.5 kJ/m^2^ when untreated to 115.6 kJ/m^2^ thereafter, while a 170 kV–129 kGy setting increased *a*_uc_ about 15%, to 83.3 kJ/m^2^, when compared to the untreated sample. Scanning electron microscopy (SEM) showed the 250 kV–86 kGy HLEBI increases KF/PP adhesion with increased consolidation and KF bundling, while the electron spin resonance (ESR) showed HLEBI generates dangling bonds (DBs) in KF and PP, which is evidence of the strengthening KF/PP interface. X-ray photoelectron spectroscopy (XPS) of the N1s spectrum of Kevlar fiber from the fracture region of the untreated sample showed a dominant peak at 399.5 eV with 82.7% area, which is characteristic of the Kevlar backbone N–(C=O)–, indicating poor adhesion with fiber pullout. However, the dominant peak was shifted in the 250 kV–86 kGy sample to that of strongly bonded imines, –C=N–, at 398.6 eV and 36.8%, indicating strong bonds generated at the KF/PP interface. Together, the N1s, C1s and O1s spectra indicate increased polar groups, reduced weak Van der Waals forces, and the generation of a strong active nitrogen-containing interphase, acting to reduce fiber pullout to increase the impact strength of the [PP]_5_[KF]_4_ composite system.

## 1. Introduction

**Figure 1:**
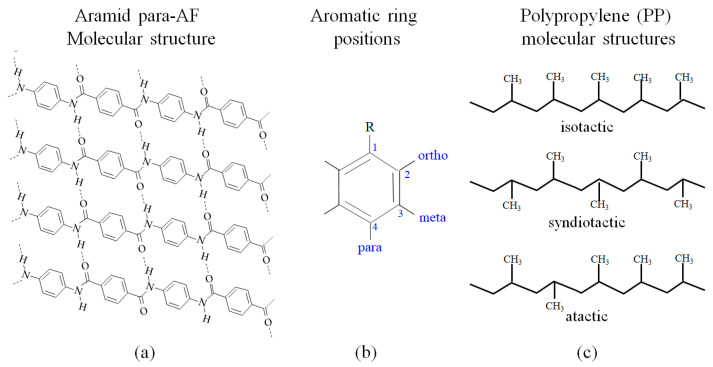
Constitutional formula of *para*-aramid (AF) (**a**); designations of the carbon atoms sequentially around aromatic ring (**b**); and PP molecular structures (**c**).

Kevlar^®^ is a trade name for *para*-aramid fiber (*para*-AF), whose chemical name is poly(p-phenylene terephthalamide) (PPTA), which is a variant of AF that was first developed by DuPont in 1965. Kevlar has long been utilized for articles that demand high thermal insulation, high impact strength, and excellent shielding from cosmic rays, which has notably been used for the Apollo astronauts’ space suits used for walks on the moon [1]. Another widely used *para*-aramid is Twaron^®^, which was fabricated by Teijin Aramid. In 2025, the global market-size value of *para*-aramid was reported to be about USD 3.5 billion and forecasted to be about USD 7 billion by 2033 [2], increasing each year by 5–10% [3]. The *para*-aramid fiber is synthetic, having a molecular structure of aromatic rings connecting alternately to either two −NH groups or two −CO groups in a *para* configuration at positions 1 and 4 around the aromatic rings as illustrated in Figure 1a,b. Typically, Kevlar (density, *ρ* of 1440 kgm^−3^) [4] is 20% lighter than carbon fiber CF (1800 kgm^−3^) [5] and an order of magnitude stronger than aluminum [4].

As shown in Figure 1a, *para*-AF has polymer chains highly oriented along the longitudinal axis, demonstrating a higher proportion of chemical bonds to take on the load, and thus it has a high ultimate tensile strength (UTS), which is typically reported as 3.6 GPa [4]. For comparison, the UTS of other high-strength reinforcements range from 4.3 to 6.0 GPa for carbon fiber (CF) [6,7] and from 1.7 to 3.4 GPa for glass fiber (GF) [6]. The highly oriented structure allows *para*-AF to exhibit good heat resistance with an exceptionally high peak melting point, which is reported as 536.7 °C [8]. Kevlar has superior thermal stability, excellent thermal and electrical insulation, and creep resistance due to this conjugated structure of π bonds along the chain axis [2,9,10,11,12].

Drawbacks to Kevlar include poor adhesion and low miscibility with other polymers due to their typically smooth surface and the insufficiency of active chemical groups [13,14], and being hydrophilic, resulting in a high degree of water absorption that can lower mechanical properties, such as impact in polymer composites [15]. Also, Kevlar has high UV-ray sensitivity, poorer compression strength than CFs, and difficulty in cutting, resulting in fraying [16].

However, Kevlar has a successful track record for several applications in various areas and products, including aerospace, automotive, train transportation, marine cordage, marine hull reinforcement, safety equipment, bulletproof vests, batteries, and flame-resistant suits and gloves as well as an asbestos substitute [11,12,16,17,18]. Another well-known AF variant is *meta*-AF, or Nomex^®^, with linkages at positions 1 and 3, thus having a zig-zag pattern. Although not as strong as *para*-AF in the axial direction, Nomex has good flexibility along with exceptional flame resistance, abrasion resistance, chemical resistance, and thermal stability. Despite *meta*-AF exhibiting three to seven times lower mechanical strength than *para*-AF [16] and more free volume [19], *meta*-AF and *para*-AF are often combined to optimize the properties of flame resistance, thermal stability, and flexibility along with strength to maximize safety [1].

Since this paper focuses on Kevlar, recent research on *para*-AF is covered here, including the following: treatment of *para*-AFs themselves [11,16,17,20,21,22,23,24,25,26,27,28,29,30] and recycled *para*-AF [24,31] for better performance; increasing flame resistance [24,26,32]; enhancing UV resistance for higher strength [30]; use in aerogels [25,26]; improving properties of *para*-AF paper [27,28,29,33]; employing for EV battery separators [34]; use in hybrid textiles [31]; intelligent shear-thickening fluid for bullet-proof clothing [35]; lightweight material for honeycomb sandwich structures [36]; *para*-AF/polymer composites [21,24,27,32,33,36,37,38,39,40,41,42]; and hybrid polymer composites such as KF-CF/polymer [37,40]; and KF-basalt fiber (BF)/polymer [40,41,42].

Specifically, common treatments for *para*-AF composites have included the addition of particles such as nano-silica [21], graphene oxide (GO) [36], and multi-walled carbon nanotubes (MWCNTs) [22] to enhance *para*-AF/matrix adhesion along with *para*-AF itself. Nano-silica particles have been added to Kevlar laminates with polyester elastomer addressing challenges with shear thickening [21]. For honeycomb CFRP/KF/PP/KF/CFRP sandwich structures, GO has been successfully grafted onto KF using polydopamine (PDA) as an adhesion medium, substantially improving the bonding strength of the CFRP panel to a PP honeycomb core with peak force and energy absorption increased 70.0% and 109.4%, respectively [36]. Poly-p-paraphenylene terephthalamide (PPTA) oligomer-modified multi-walled carbon nanotubes (MWCNTs) have been grafted on *para*-AF, resulting in a significant increase in the tensile properties of a *para*-AF single fiber test [22]. Other treatments have included DMSO/KOH treatment to split the KF into extremely fine nano-KF for high dispersion [17]; spinning methods to obtain *para*-AF nano diameters with narrow distribution between 20 and 25 nm [23]; using *para*-AF recycled by phosphoric acid as an improved flame retardant for thermoplastic polyurethane (*para*-AF/PU) [24]; and making antibacterial *para*-AF by depositing silver ions to surface, protecting 99% against *Escherichia coli* (*E. coli*) and *Staphylococcus aureus* (*S. aureus*) [20].

For other treatments, an addition of *para*-aramid nanofibers (PANFs) has strengthened polyimide (PI) aerogels [25]; and *para*-AF/graphene pulp has increased aerogel flame resistance [26]. For nano-KF for paper, ample research exists, including strengthening *para*-*para*-AF/polyethylene sulfide (PPS) paper by grinding *para*-AF into ultrafine pulp [27]; plasma-activated interfacial precision for *para*-AF papers [29]; a multi-strategy method for increasing the interfacial bonding strength of *para*-AF paper [28]; and nano *para*-AF pulps for *para*-AF paper treated with DMSO/KOH [17]. In addition, for EV battery use, KF for poly(vinylidene fluoride-co-hexafluoropropylene) (PVDF-HFP) battery separators has been shown to reduce lithium dendrite formation in order to increase the safety and performance [34]. Recent research for bulletproof clothing includes Kevlar impregnated with mesoporous silica-carbon nanotube (CNT) ionic shear thickening fluid (STF) (CNTs/STF/Kevlar) as ana intelligent material that could stop projectiles up to 110 m/s velocity [35].

We employ a PP matrix in this paper, since it is a widely used thermoplastic (TP) that is recyclable; hence, it can be repeatedly heated and solidified to reduce waste, landfill, and ocean pollution. Induced by the nanoscale spaces between methyl (–CH_3_) groups in the molecular structure, PP has a low specific gravity for reducing fuel consumption when used for gravity-bound vehicles. PP is inexpensive, and it has a solidification term tremendously shorter (about 10 times less) than that of epoxy, which helps lower energy consumption to lower the carbon footprint. The way PP is produced is via chain-growth polymerization from the monomer propylene. The PP is partially crystalline and non-polar, and it is a white, mechanically rugged material that has a high chemical resistance [43,44,45,46,47]. The –CH_3_ groups improve mechanical properties and thermal resistance, although chemical resistance decreases [48,49]. Properties depend on the molecular weight and molecular weight distribution, crystallinity, type and proportion of co-monomer and isotacticity, as shown in Figure 1c [48]. These allow PP to be used as a durable engineering plastic for gears, automobile parts and computers, since it exhibits high resistance to gasoline, elastic deformation, relaxation, creep and heat less than 260 °C. It also exhibits low thermal expansion and dimensional stabilization.

There has been high interest in KFRPP composites due to their high impact resistance, high tensile strength, toughness, high thermal stability, lightweightness, low cost of PP, and ease of fabrication [33,37,38,39,40,41,42,50]. Studies showed that 3D-angle interlock KFRPP composites exhibited a higher peak load, higher energy absorption, and lower cone formation at the back of the panel during low velocity impact than 2D-plain woven and 3D-orthogonal composites due to their higher in-plane stiffness [33]; maleic anhydride grafted to PP enhanced high strain rate behavior [39]; and AF has been modified with ammonium sulfate for AF/PP ethylene diene monomer composites [38]. However, KFRPP displayed more difficult end milling than CFRPP or KF-CFRPP interyarn hybrids [37]; and laser machining (drilling 11.6 mm holes) of KF-129/PP and KF-129/polyetherimide (PEI) composites of 16, 24, and 30 layer thicknesses showed PP has slower flow than PEI and remains as residue at the laser site [50].

The hybridization of Kevlar with other fibers, such as interwoven KF-CF weaves, has been receiving much attention due to their strength over those of just KF [37]. For 2D laminates, KFR-basalt fiber (KF-BFRPP) exhibited higher tensile and in-plane compression behavior, elastic modulus, strength, and fracture strain than either KFRPP or BFRPP [40]; the 3D angle interlock KF-BFRPP composite exhibited improvements in tensile behavior but no apparent increase in in-plane compression strength compared with KFRPP or BFRPP [41]; while for low-velocity drop weight impact, BFRPP exhibited the highest peak force, but KF-BFRPP apparently had highest energy absorption [42].

It follows that the homogeneous low potential electron beam irradiation (HLEBI) of the 100 kV class has a proven track record of improving the properties of several materials [7,51,52,53,54,55,56,57]. HLEBI treatment often induces hardening, high wear resistance, mist resistance, and sterilization in polymers [51,52], and it can raise the electrical conductivity of polymers such as Teflon^®^ [57]. Improving the fracture toughness of fiber-reinforced polymers (FRPs) has been achieved by applying HLEBI to sample surfaces [53,58] and to fibers directly prior to assembly and fabrication to increase the mechanical properties of CFRP [53,54,55,56,59]. HLEBI generates DBs, i.e., lone pair electrons that work three ways. One is by electrical repulsion creating nano-internal compressive stress to strengthen materials, crosslinking, and activating surfaces for enhanced adhesion, particularly of hard-to-adhere components such as TP and CF [7,54,55]. HLEBI has been found to increase the impact value in other FP-PP composites such as CFRPP [60].

The Charpy impact test was selected because it is used as a primary test to evaluate candidate structural materials used in airplanes, space vehicles, and automobiles to provide information on reaction and resistance to high-velocity impact [5]. Charpy impact is a drop weight pendulum test to determine impact absorption mechanisms and is used to evaluate the impact toughness of materials for quality control (QC). Charpy can be used as a quick and inexpensive way to assess reactions to high velocity impact. This is to screen new candidate materials for aircraft or automobiles for possible extensive testing, such as tensile, compression, interlaminar shear, fatigue, aging, and environmental factors such as ultraviolet or cosmic rays, temperature and humidity [5].

This paper focuses specifically on impact strength, since it is one of the most important properties for Kevlar composites. Moreover, as far as the authors know, this appears to be the first study in the literature on electron beam (EB) to KF-polymer (KFRP) composites. Studies on EB have been found for treatment of the KF component, but these are sparse [61,62]. One study found that a strong 1000-kV class EB of 1200 keV at a 150 kGy dose was optimal for grafting acrylic acid onto a KF surface [61], while another found that 250 kV–450 kGy EB improved the dyability of KF for clothing [62]. Advantages of HLEBI include that it uses no chemical treatments, does not produce waste, and takes a short time. Therefore, to fill the research gap, the novel part of this paper is to investigate and demonstrate that the 100 kV-class HLEBI can increase the Charpy impact strength of KFRP, namely a recyclable TP-containing KFRPP composite, by increasing the important property of impact strength to 50%. This can assist with reaching Sustainable Development Goals (SDGs) for a cleaner environment of our Earth.

## 2. Materials and Methods

### 2.1. Composite Fabrication

**Figure 2:**
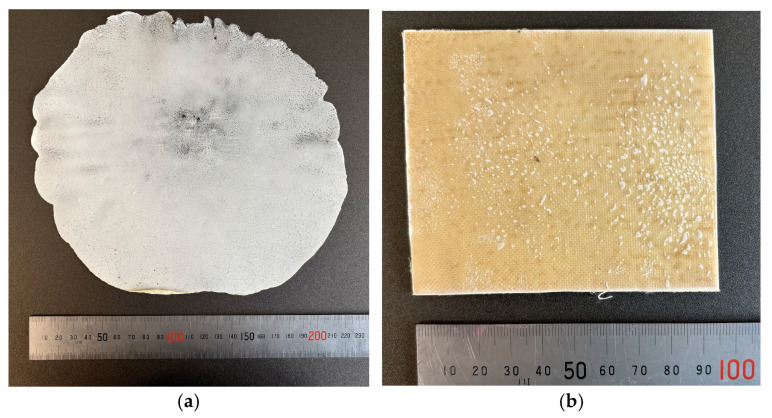
Photographs of (**a**) a single PP sheet and (**b**) pre-complexed KFRPP laminated composite.

**Figure 3:**
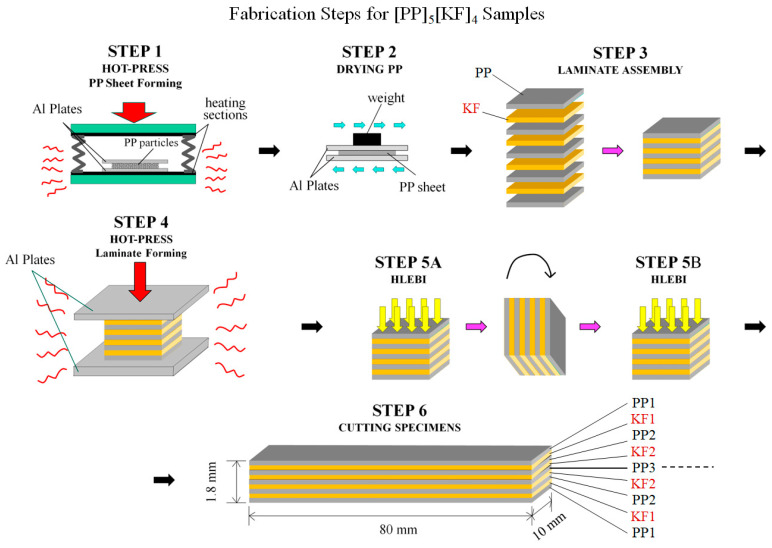
Schematic of fabrication steps for [PP]_5_[KF]_4_ samples. Ply designations from outer surfaces are shown.

Composite fabrication is illustrated in Figure 2 and Figure 3. The KFRPP samples were constructed with sized KF fabric (Kevlar^®^ 29; DU PONT-TORAY Co., Ltd., Tokyo, Japan). The sizing formulation is proprietary. Thermoplastic (TP) polypropylene (PP) sheets, BC06C, were provided by Novatec, Nissho Ltd., Tokyo, Japan. As illustrated in Figure 3, there were 6 basic fabrication steps:

**STEP 1**: PP sheets (plies) with dimensions approximately 200 μm in thickness were formed with a hot press by PP particles (3 g) under 16 MPa at 493 K for 3 min, as shown in the photo in Figure 2a.

**STEP 2**: PP plies were dried by air convection between Al plates secured by a weight.

**STEP 3**: Laminate assembly was carried out in an interlayered configuration of 4 sized KF plies between 5 PP sheets with layup [PP1-KF1-PP2-KF2-PP3-KF2-PP2-KF1-PP1] designated here as [PP]_5_[KF]_4_. This is so the thickness conforms to Japanese Industrial Standard JIS K 7077 of 2.0 ± 0.2 mm [63].

**STEP 4**: Panels were then solidified by a one-directional hot press (IMC-185A, Imoto Machinery Co., Ltd., Tokyo, Japan) at 4.0 MPa and 493 K for 3 min to form the laminates.

**STEP 5 (A and B)**: Panels were then HLEBI treated on both sides (see the next section).

**STEP 6**: Samples were cut to size to a length, width and thickness of 80 × 10 × 1.8 mm. When ply thicknesses are estimated to be equal in the finished specimens, they are calculated to be 1800 μm/9 = 200 μm.

HLEBI-treated samples were compared to a control of untreated without HLEBI (eliminating Step 5). The volume fraction *V*_f_ of the KFs was 0.55.

### 2.2. Condition of HLEBI

**Figure 4:**
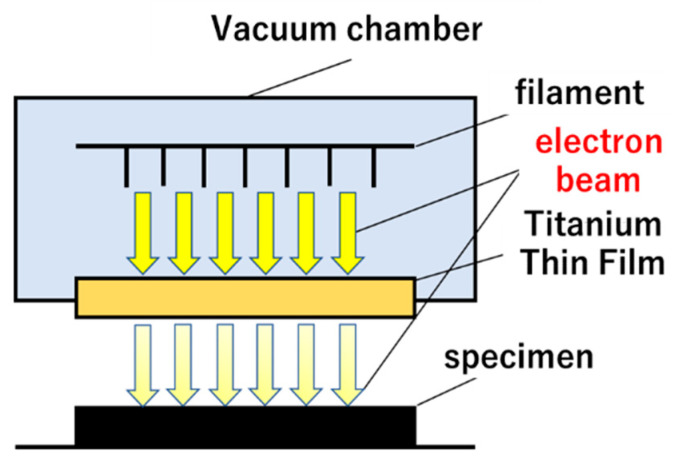
Schematic equipment of electron beam irradiation processor.

A new treatment for KFRPP samples of HLEBI on both sides was carried out using an electron-curtain processor (Type CB175/15/180L, Energy Science Inc., Woburn, MA, USA, Iwasaki Electric Group Co., Ltd., Tokyo, Japan) after lamination assembly and hot-press [12,13,14,15,21,22,23,24,25,26,27,28,29,30,31,32,33,34,35]. Figure 4 shows a schematic of the HLEBI processor. Samples were homogeneously irradiated by the linear electron beam gun with low energy through a titanium thin film window attached to a vacuum chamber of 240 mm diameter. A tungsten filament in a vacuum was used to generate the electron beam at low energy (cathode potential setting, *V*_c_: kV), at 170 kV or 250 kV (see description below), and at an irradiating current density (I, A/m^2^) of 0.089 A/m^2^.

The electron beam is generated in a vacuum using a Ti filament. The distance between the sample and the window is set to be 25 mm. To prevent oxidation, the samples are kept in a protective atmosphere of nitrogen gas with a residual concentration of oxygen below 300 ppm. The constant flow rate of nitrogen gas was set to be 1.5 L/s at the constant nitrogen pressure condition of 0.1 MPa. Each irradiation dose (43.2 kGy) was applied for only a short time (0.23 s) to avoid excessive heating of the sample; the temperature of the sample surface remains below 323 K just after irradiation, which was confirmed for all samples by the emission pyrometer measurements. The sample in the aluminum plate holder (0.15 × 0.15 m^2^) is transported on a conveyor at a speed of 10 m/min. The sheet electron beam irradiation is applied intermittently; one sweep going one way is 43.2 kGy. Repeated irradiations to both side surfaces of the samples are used to increase the total irradiation dose. The interval between sweeps was 30 s. The irradiated dosage was proportional to the irradiation current (*I*, mA) and number of irradiations (*N*), whereas it was inversely proportional to the conveyor speed (*S*, m/ min).

The irradiation dose has been controlled by the integrated irradiation time in each of the samples. Here, the irradiation dose is corrected by using an FWT nylon dosimeter of RCD radiometer film (FWT-60-00: Far West Technology, Inc., 330-D South Kellogg, Goleta, CA, USA) with an irradiation reader (FWT-92D: Far West Technology, Inc., 330-D South Kellogg Goleta, CA, USA). The dose is 0.0432 kGy for each irradiation sweep.

Impact strength optimization tests according to HLEBI *V*_c_–dose setting combinations were carried out for the [PP]_5_[KF]_4_ with 3 specimens each, as shown in the test matrix in Table 1. This was to conserve the limited amount of material available. Out of the data sets tested, 170 kV–129 kGy, and 250 kV–86 kGy (underlined) yielded the highest improvement in impact strength, *a*_uc_ over untreated (see Section 3.3). Therefore, as indicated in Table 1, additional specimens were tested, totaling 14 each for the 170 kV–129 kGy and 250 kV–86 kGy conditions, respectively. This was to have the number of specimens prepared and tested to be more than 10 for experimental validity in conformity with JIS K 7077 for Charpy impact tests [63].

### 2.3. Penetration Depth, D_th_ of HLEBI

Based on the mean density (*ρ*: kg/m^3^) and specimen surface electrical potential [*V*_x,y_], the penetration depth (*D*_th_:/m) of HLEBI is expressed by the Christenhusz–Reimer equation [64]:

*D*_th_ = 66.7[*V*_x,y_] ^5/3^/*ρ* (1)

Here, subscript ‘*x*’ is the *V*_c_ setting, and ‘*y*’ is the surface entered. When calculating for a *V*_c_ of 170 kV for the surface of the outer PP1 ply, ‘*x*’ is 170 and ‘*y*’ is PP1; thus, [*V*_x,y_] is designated [*V*_170(PP1)_]. Using the principal form of Equation (1), [*V*_170(PP1)_] is mainly dropped by the Ti window (Δ*V*_Ti_) and N_2_ gas atmosphere (Δ*V*_N2_) of the HLEBI processor. Thus, when *V*_c_ is set to 170 kV, [*V*_170(PP1)_] is calculated as follows [5,64]:

[*V*_170(PP1)_] = 170 kV − Δ*V*_Ti_ − Δ*V*_N2_ (2)

Equations (3) and (4) show the calculations for Δ*V*_Ti_ and Δ*V*_N2_ using a 10 μm thickness of the titanium window (*T*_Ti_, homogeneous density assumed to be constant value of 4540 kgm^−3^) and the 25 mm distance between the sample and window (*T*_N2_) in the N_2_ gas atmosphere (density: *ρ*_N2_ = 1.13 kgm^−3^) [64]:

Δ*V*_Ti_ = *T*_Ti_/*D*_thTi_ × 170 kV = *T*_Ti_*ρ*_Ti_/[66.7 × (170 kV)^2/3^]       = (10^−5^ m) × (4540 kgm^−3^)/[66.7 × (170 kV)^2/3^] = 22.2 kV (3)

Δ*V*_N2_ = *T*_N2_/*D*_thN2_ × *V*_Ti_ = *T*_N2*ρ*N2_/[66.7 × (*V*_Ti_)^2/3^]                              = (25 × 10^−3^ m) × (1.13 kgm^−3^)/[66.7 × (170 − 22.2 kV)^2/3^] = 15.1 kV (4)

Since the dropped potential values were 22.2 kV and 15.1 kV, *V* is calculated as shown below:

[*V*_170(PP1)_] = 170 kV − 22.2 kV − 15.1 kV = 132.7 kV (5)

When substituted in Equation (1) using a PP density of 900 kgm^−3^ [65], the calculated *D*_th_ into outer ply PP1 is 256 μm, which is greater than its 200 μm ply thickness. Hence, the PP1 ply is activated from the specimen surface and throughout the thickness to generate vacant sites with dangling bonds (DBs) [54,55]. Table 2 lists *D*_th_ values into PP1 for *V*_c_ settings of 170 and 250 kV, along with their Δ*V*_Ti_, Δ*V*_N2_, and *V*_x(PC1)_ values, respectively from Equations (1)–(5), illustrating that *D*_th_ increases with *V*_c_.

The HLEBI activation will then penetrate the PP1/KF1 interface into the next ply, AF1, and the *D*_th_ is calculated using [*V*_170(AKF1)_] and KF density with a *ρ* of 1440 kgm^−3^ [4]. In this way, the *D*_th_ can be calculated successively throughout the layered sample thickness (see the Discussion section). Note, it is assumed here that *D*_th_ is independent of the HLEBI dose (kGy).

### 2.4. Charpy Impact Test

To evaluate the impact fracture toughness, Charpy impact values (*a*_uc_) of the KFRPP with and without HLEBI were measured using a standard impact fracture energy measurement system (Shimadzu Corporation No.51735, Tokyo, Japan) in accordance with JIS K 7077 [63].

The Charpy impact test is used to determine the energy required to fracture a specimen piece using the force of a hammer pendulum. When *W*, *R*, *α*, *β*, *b* and *t* are the hammer weight (N), hammer turning radius (0.21 m), Hammer swing angle (deg), angle of swing after impact (deg), specimen width (mm) and a specimen thickness (mm), respectively, the impact fracture energy *E* (kJ) is expressed by the following equation [63]:

*E* = *W R* [(cos *β* − cos *α*) − (cos *α*′ − cos α) (*α* + *β*)/(*α* − *α*′)] (6)

Here, *W*, *R*, β, α and α′ are the hammer mass (0.86 kg); length (m) of the hammer weight point from the rolling center; the maximum angle after impact (Radians); the start angle before impact (*α* = 132 deg or 2.3 Radians); and the maximum angle of the blank test, respectively.

Three blank tests are conducted to calibrate the impactor for atmospheric conditions such as atmospheric pressure, temperature, and humidity. The Charpy impact value *a*_uc_ (kJm^−2^) is expressed by the following equation [63]:

*a_uc_* = *E*/(*b t*) (7)

Here, *E*, *b* (=10 ± 0.2 mm) and *t* (=1.5 ± 0.15 mm) are the impact fracture energy (J), sample width (mm), and thickness (mm). The distance between supporting points in the specimen holder was 40 mm.

Evaluating the probability of fracture (*P*_f_) has been a convenient method of quantitatively analyzing experimental values relating to fracture, which are often used in industry to determine manufacturing reliability in quality control (QC). *P*_f_ is expressed by the following equation, which is a generalized form of the median rank method [65]:

*P*_f_ = (*I* − 0.3)/(*N*_s_ + 0.4) (8)

where *N*_s_ and *I* are the total number of samples and the ascending strength order of each sample, respectively. Impact tests were carried out 30 ± 0.5 h after HLEBI treatment.

### 2.5. Scanning Electron Microscopy

A tabletop SEM Model JCM-6000 Plus Neoscope (JEOL, Ltd., Tokyo, Japan) was used to analyze the fracture surfaces of the samples. Quantitative statistical image analysis on fracture surfaces was not carried out. However, SEM observations were conducted over multiple regions across the fracture surfaces of untreated and HLEBI-treated KFRPP specimens, and consistent differences in the morphology and KF/PP interaction were observed. Analysis such as surface mapping to obtain the PP bonding area fraction would be interesting for future work.

### 2.6. Electron Spin Resonance (ESR) Analysis

To obtain more precise information on atomic-scale structural changes in the KF and PP, the DB density was obtained using an electron spin resonance spectrometer (ESR, JES-FA2000, Nippon Denshi Ltd., Tokyo, Japan) [53]. ESR is utilized to detect unpaired electrons by their spins (*m*_s_ = +/−1/2), since electrons have a magnetic moment and spin quantum number. The unpaired electrons’ magnetic moments either align themselves parallel or anti-parallel to an induced magnetic field, producing a peak at a particular magnetic field, *B*. The microwave frequency range used in the ESR analysis was the X-band at 9.45 ± 0.05 GHz with a field modulation of 100 kHz. The microwave power was 1 mW. The magnetic field *B* was varied from 317 to 327 mT. The spin density was calculated using a Mn^2+^ standard sample. Based on the standard calibration material TEMPOL(2,2,6,6-tetramethyl-4-piperidinol-1-oxyl) and Mn^2+^ in the MnO, only ESR spectra, instead of spin densities, were given, since relative peak intensities enable conveniently comparing the number of DBs (spin density) generated in the samples from the HLEBI [51,66].

### 2.7. X-Ray Photoelectron Spectroscopy (XPS) Analysis

XPS tests were conducted on the Kevlar fiber surface at the sample fracture regions using a Model PHI Quantera II XPS instrument from ULVAC-PHI, Inc., Chigasaki, Kanagawa, Japan. For peak analysis, khervefitting software version 1.8, March 2026 was used with charge corrections applied based on the Kevlar aromatic C–C literature value of 284.6 eV [67].

## 3. Results

### 3.1. Effects of HLEBI on Charpy Impact Value of [PP]_5_[KF]_4_ (Kevlar) Composite

**Figure 5:**
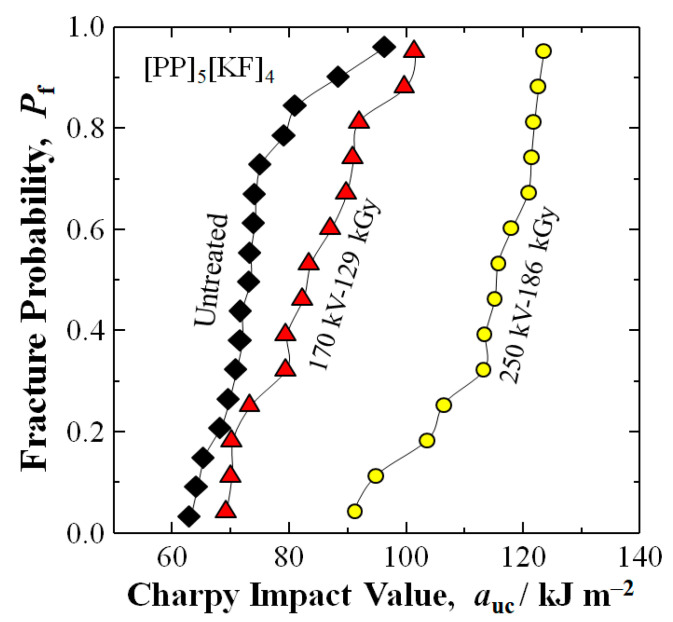
Changes in *a*_uc_ of [PP]_5_[KF]_4_ untreated and treated by optimal *V*_c_-dose combinations of 170 kV–129 kGy and 250 kV–86 kGy against *P*_f_.

Figure 5 shows the experimental results of a [PP]_5_[KF]_4_ Kevlar composite untreated and treated by an optimal HLEBI of 250 kV–86 kGy and 170 kV–129 kGy, respectively, where the Charpy impact value *a*_uc_ is significantly strengthened at all *P*_f_. The 250 kV–86 kGy condition increases *a*_uc_ at a median fracture probability (*P*_f_ of 0.50) 59% from 72.5 kJ/m^2^ for untreated to 115.6 kJ/m^2^, while 170 kV–129 kGy increases *a*_uc_ about 15% to 83.3 kJ/m^2^. The *a*_uc_ increase by 250 kV–86 kGy is 40% higher than that by 170 kV–129 kGy.

### 3.2. Parameter Weibull Analysis

The two-parameter Weibull coefficient (*n*) is one of the standard parameters for comparing structural materials [68,69,70] and is often used in quality control (QC). When *a*_uc_ represents the experimental Charpy impact values that are constants, the fracture probability (*P*_f_) as a function of risk of rupture (*a*_uc_/*a*_o_) is expressed as [68,69,70]

*P*_f_ = 1 − exp[−(*a*_uc_/*a*_o_)^n^] (9)

and rearranged in its linear form:

ln[−ln(1 − *P*_f_)] *n*ln*a*_uc_ − *n*ln*a*_o_ (10)

**Figure 6:**
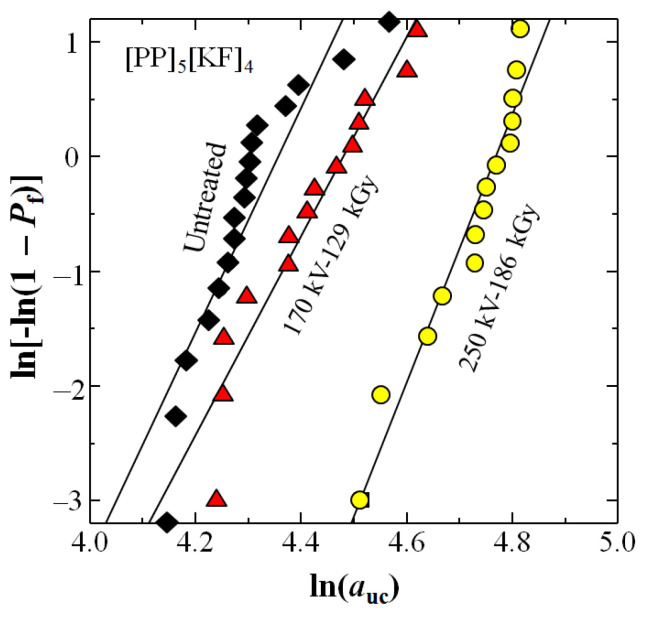
Two-parameter Weibull plots from the data in Figure 5.

Figure 6 shows two-parameter Weibull plots from Equation (10) for the three data sets of Figure 5. Table 3 shows that the 250 kV–86 kGy HLEBI increases the Weibull modulus (slope) *n* 20% from 9.76 for untreated to 11.67, while the 170 kV–129 kGy reduces *n* 10% to 8.71.

### 3.3. Average a_uc_

**Figure 7:**
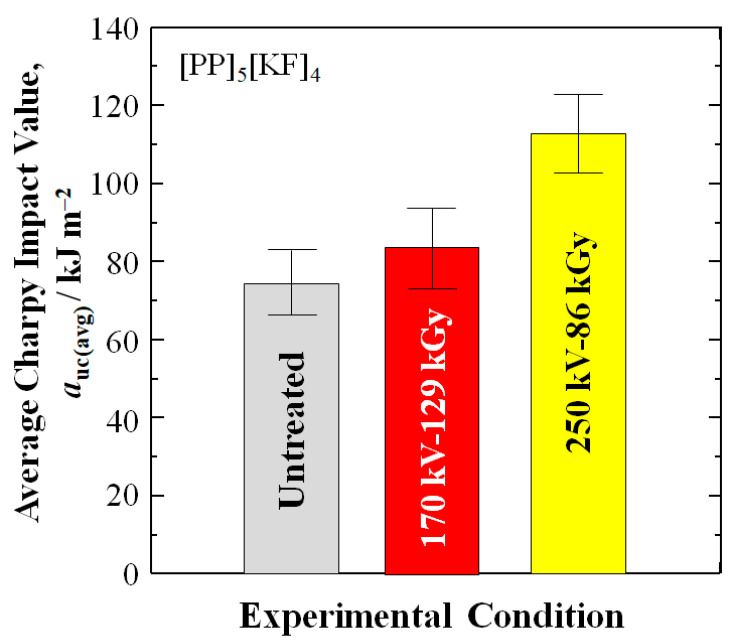
Graph of average *a*_uc_ and standard deviations for the data sets in Figure 5.

Table 3 and Figure 7 show that the average *a*_uc_ value of the 250 kV–86 kGy data set is increased 52% over untreated from 74.3 to 113.0 kJm^–2^, while the standard deviation for the 250 kV–86 kGy samples is higher (10.2) than untreated (8.4). In addition, Figure 7 shows the 250 kV–86 kGy minimum standard deviation bar is above the maxima for both untreated and 170 kV–129 kGy and untreated data sets.

**Figure 8:**
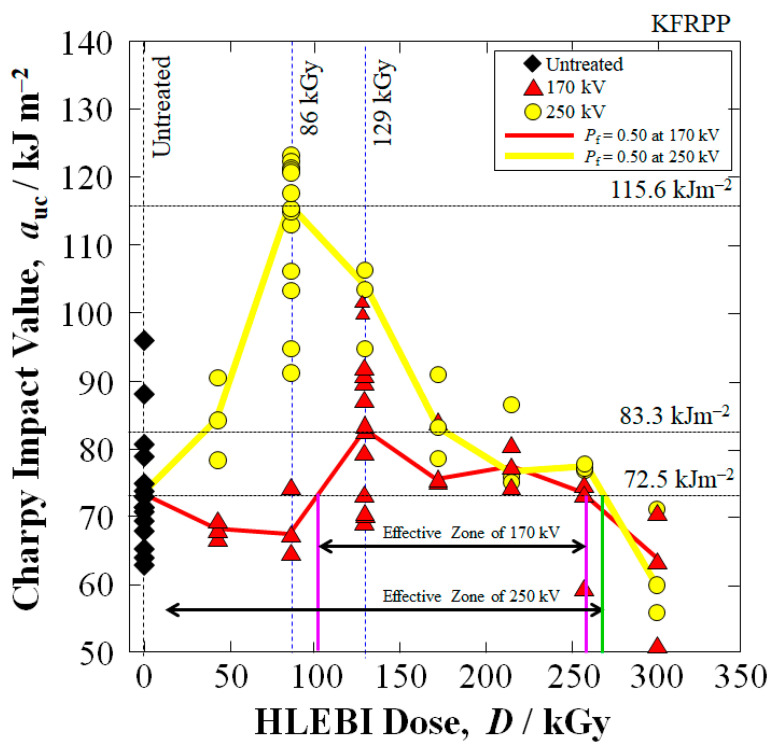
Effects of HLEBI irradiation dose (kGy) on experimental impact values (*a*_uc_) for KFRPP ([PP]_5_[KF]_4_) samples untreated and treated by 170 and 250 kV HLEBI to obtain an optimum dose for impact strength. Effective zones for increasing *a*_uc_ are indicated for 170 and 250 kV conditions, respectively.

Figure 8 shows impact strength optimization test results conducted experimentally, that were utilized to obtain optimal *V*_c_-dose combinations in Figure 5. The 170 kV–129 kGy and 250 kV–86 kGy data sets exhibit the highest impact values. On the other hand, higher doses above 172 kGy at both 170 and 250 kV HLEBI tend to decrease *a*_uc_ with 301 kGy decreasing *a*_uc_ significantly more than the untreated samples. This is due to excess dangling bonds and chain scission by the excess HLEBI dose. Hence, as shown in Figure 8, out of the HLEBI doses tested, the optimum HLEBI settings for increasing the impact strength of the Kevlar fiber [PP]_5_[KF]_4_ composite is considered to be 250 kV–86 kGy (yellow curve). For the 170 kV HLEBI (red curve), the optimum dose appears to be 129 kGy, albeit *a*_uc_ is considerably lower than that for 250 kV–86 kGy. Note the optimums are at different doses 86 and 129 kGy, which could be an effect of balance between the *D*_th_ (related to *V*_c_) and DB density (related to dose), whose analysis is beyond the scope of this paper. In all cases, HLEBI was applied to the finished surfaces of the specimen.

It was suggested that multi-factor (DOE) or response surface analysis be carried out, which have been valuable for optimizing multi-factor processes in manufacturing for cost and performance. These were not implemented here; however, since the primary objective was to experimentally determine whether HLEBI can enhance the impact strength of the KFRPP and to identify successful conditions within the processing window available. Because this is the first reported observation of HLEBI increasing the impact strength of KFRPP, the study was designed as an exploratory investigation. It is possible that higher cathode voltages or increased the fine-tuning of doses may increase *a*_uc_ further, but unexpected physical and chemical transitions in the KFRPP may occur, which would need to be taken into account. Moreover, in using the recyclable PP for scaled-up process, to obtain a full picture of strength and cost, along with effect on the environment and human working conditions, life cycle assessment (LCA) would be highly necessary.

**Figure 9:**
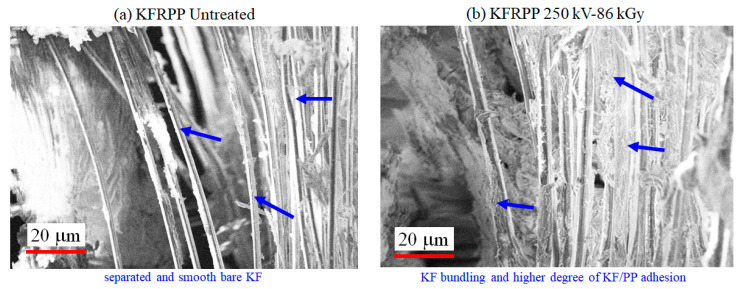
SEM photomicrographs of fractured KFRPP impact samples (**a**) untreated and (**b**) treated by 250 kV–86 kGy HLEBI.

**Figure 10:**
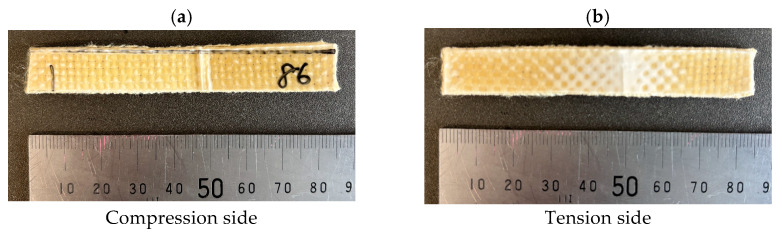
Photos of a typical fractured [PP]_5_[KF]_4_ Charpy impact sample. Shown are 250 kV–86 kGy compression (**a**) and tension (**b**) sides.

Figure 9a,b show SEM photomicrographs of fractured KFRPP impact samples untreated and treated by 250 kV–86 kGy HLEBI. The 250 kV–86 kGy HLEBI exhibits increased KF/PP adhesion with increased consolidation and bundling of the KFs (blue arrows). On the other hand, untreated exhibits poor KF/PP adhesion with separated fibers and smooth bare fiber surfaces. Most KFs in the SEM photos are observed to have diameters (*d*_f_) of about 1 to 2 μm. Figure 10 shows the typical fractured Charpy impact specimens.

## 4. Discussion

### 4.1. Action of HLEBI Penetration Depth for Strengthening

The Christenhusz–Reimer equation (1) has been used to calculate *D*_th_ from the surface electrical potential for homogeneous materials [64]. However, it seems logical that *D*_th_ can be calculated for each successive ply going deeper into sample thickness even if they are different materials [5]. Equations (11) and (12) show *D*_th_ calculations for each medium the HLEBI passes through when *V*_c_ is 170 kV: the Ti window, N_2_, PP1 and KF1, while Table 4 lists the *D*_th_ values for 170 and 250 kV. Consequently, Table 4 shows that as *V*_c_ is increased from 170 to 250 kV, *D*_th_ into the next deeper KF1 ply goes from 13 to 192 μm (bold underlined). The strengthening of *a*_uc_ at 250 kV can be explained by HLEBI activation throughout a greater portion of the 200 μm KF1 ply thickness, with 192 μm/200 μm, or 96% activation. For PP1 outer ply, Table 4 shows full penetration through the 200 μm thickness: 256 μm and 602 μm for 170 and 250 kV, respectively.

To obtain *D*_th_ into the next ply KF1, [*V*_170(KF1)_] KF density (1440 kgm^−3^) [4] is used to calculate Δ*V*_PP1_ and *V*_170(KF1)_ as in Equations (1)–(5) [64]:

Δ*V*_PP1_ = *T*_PP1_/D_thPP1_ × *V*_N2_ = *T*_PP1_*ρ*_PP1_/[66.7 × (*V*_N2_)^2/3^]                                            = (20.0 × 10^–5^ m) × (900 kgm^–3^)/[66.7 × (*V*_c_ − Δ*V*_Ti_ − Δ*V*_N2_ − Δ*V*_PP1_)^2/3^] = 104 kV (11)

*V*_170(KF1)_ = 170 kV − 22.2 kV − 15.1 kV − 104 kV = 28.9 kV (12)

Therefore, by Equation (1), using *V*_170(KF1)_ = 28.9 kV, *D*_th_ into the KF1 ply is 13 μm.

Thus, although *a*_uc_ was increased, the 170 kV HLEBI is estimated to penetrate about 6.5% through the KF1 ply (13 μm/200 μm) not reaching the KF1/PP2 interface, as shown in Table 4. However, Figure 5 shows that applying 170 kV HLEBI raised the *a*_uc_ of the PP]_5_[KF]_4_ samples over that of the untreated samples at all *P*_f_ with the *a*_uc_ at a *P*_f_ of 0.50 increased 15%.

Likewise, the 250 kV HLEBI penetrates about 96% through the KF1 ply (192 μm/200 μm), resulting in about three times more net gain in *a*_uc_ than that at 170 kV with *a*_uc_ significantly increased at all *P*_f_. The high *D*_th_ of 192 μm results in higher impact strength.

Enhancements are despite the low electrical conductivities reported for pure PP at 10^–14^ to 10^–16^ S/m [71] and (industrial) KF at 2.02 × 10^–14^ S/m (4.95 × 10^+13^ Ω·m) [72]. To reach and strengthen the KF1/PP2 interface; therefore, the conductivity of PP or KF may have been raised by DB generation. By the 250 kV HLEBI, charge transfer to the KF1/PP2 interface would have more easily occurred than in the 170 kV due to 192 μm being in closer proximity to KF1/PP2. In fact, HLEBI has been reported to raise conductivity in the polymer of polytetrafluoroethylene (PTFE, Teflon) more than two orders of magnitude apparently by generating DBs as isolated radicals acting as acceptors to carry charge [57]. Hence, it is possible that DB generation can raise conductivity in the [PP]_5_[KF]_4_ samples to strengthen the KF1/PP2 interface to raise the *a*_uc_, but more work is needed to confirm this. Nevertheless, when adjusting for optimal HLEBI dose, the higher 250 kV yielded the highest *a*_uc_ values in the data sets.

**Figure 11:**
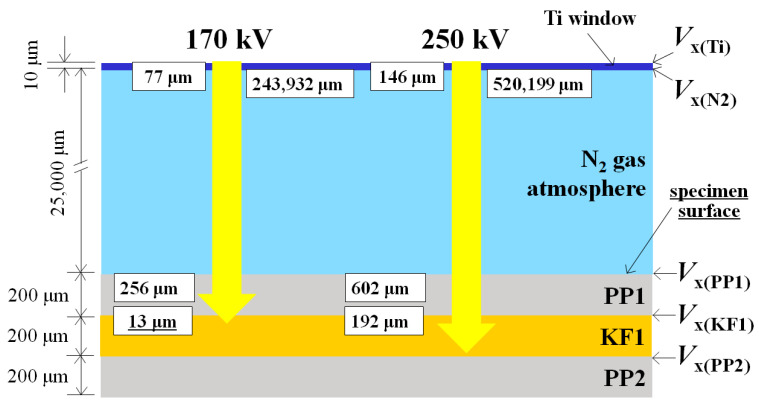
Schematic of HLEBI penetration (yellow arrows) into the surface plies of the interlayered [PP]_5_[KF]_4_ specimen cross-sections for *V*c of 170 and 250 kV data sets, respectively. *D*_th_ and *V*_x_ are shown for each interface. Only the outer three plies of one side are shown. Specimens were HLEBI-treated on both sides.

To illustrate the action of HLEBI clearly, Figure 11 shows a schematic from Table 4 of successively higher *D*_th_ into the outer plies for the HLEBI data sets. The non-homogeneity of the layered composite structure is depicted, showing PP1, KF1, and PP2 plies with locations of *V*_x_ and *D*_th_ calculated at each interface along with those of the Ti window and N_2_. Figure 8 illustrates that the increased *a*_uc_ values from 250 kV over that of 170 kV can be explained by the increased HLEBI penetration depth (*D*_th_) into the interlayered sample thickness, from 213 μm to 392 mμ, as calculated by the Christenhusz–Reimer equation [64]. The 250 kV allows deeper penetration into the second ply from the surface, KF1, from *D*_th_ of 13 μm to 192 μm, which could be from the charge possibly migrating to strengthen the KF1/PP2 interface.

Note that *D*_th_ was not determined experimentally and is considered beyond the scope of this paper; however, an experimentally measured *D*_th_ of TP is found in the literature [58]. A 0.30 MGy HLEBI-treated 280 μm thick laminated polyetheretherketone (PEEK) sheet composed of 11 PEEK films 25 μm in thickness has been carried out. After irradiation, the films were carefully disassembled, and examination by ESR indicating *D*_th_ between 250 and 280 μm was used as the drastic DB reduction zone [58].

### 4.2. Electron Spin Resonance (ESR) Results

**Figure 12:**
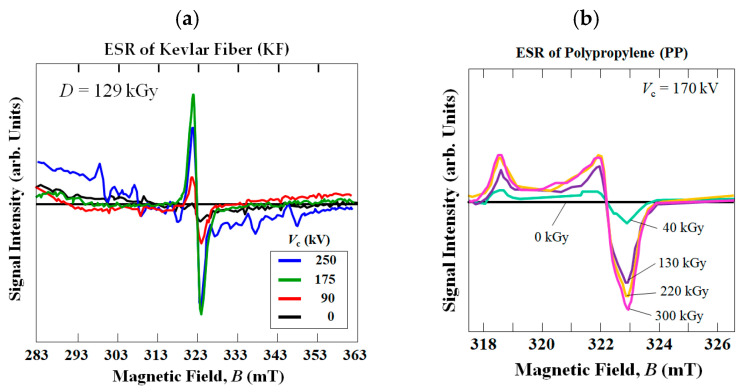
ESR signals of (**a**) Kevlar fiber (KF) and (**b**) polypropylene (PP) [46], respectively, untreated and treated by HLEBI. For KF (**a**), graphs are in black, red, green, and blue for untreated (0 kV), 90, 175 and 250 kV, respectively. For PP (**b**), graphs are in black, light green, purple, orange, and pink for 0 kGy (untreated), 40, 130, 220, and 300 kGy, respectively.

Figure 12a shows ESR results for KF untreated and HLEBI-treated at *V*_c_ values of 90, 175 and 250 kV at a 129 kGy dose. HLEBI-treated KF emits peaks with inflection points at a magnetic field *B* of 332.0 mT, while untreated KF emits a negligible peak or no peak. The peaks are generated by lone-pair electrons which emit a magnetic field [53]; therefore, DBs are generated. HLEBI-generated DBs are reported to activate the surface for increased adhesion at the fiber/matrix interface in composite materials [60]. Although Figure 12a shows that the peak intensity from 175 kV–129 kGy is higher than that at 250 kV–129 kGy, Figure 8 shows that the impact strength *a*_uc_ of the 250 kV–129 kGy samples is substantially higher. The DB density in the KF component will be lower for the 250 kV–129 kGy condition compared with that of 170 kV–129 kGy. However, the lower density DBs penetrate further into the specimen thickness regardless of homogeneity or non-homogeneity, which is the case in the interlayered structure here. Therefore, for 250 and 170 kV settings with 129 kGy dose, the penetration depth appears to be a more dominant factor in increasing *a*_uc_ than that of DB density to raise the impact strength.

Based on the ESR peaks in Figure 12b [55], HLEBI generates DBs in PP as well with peak inflection at 322.5 mT. Before HLEBI, a peak is not detected in PP. The dissociation energies of PP groups are reported to be 369 kJmol^−1^ for CH_2_-CH_2_ and 427 kJmol^−1^ for H-CH [73,74], which are apparently low enough to generate the ESR peaks. Therefore, HLEBI generating DBs in both KF and PP can explain the increased adhesion by lone pair electrons at the KF/PP interface apparently acting to raise the impact strength of the [KF]_5_[PP]_4_ system.

### 4.3. XPS Results and Nano-Strengthening Mechanism

**Figure 13:**
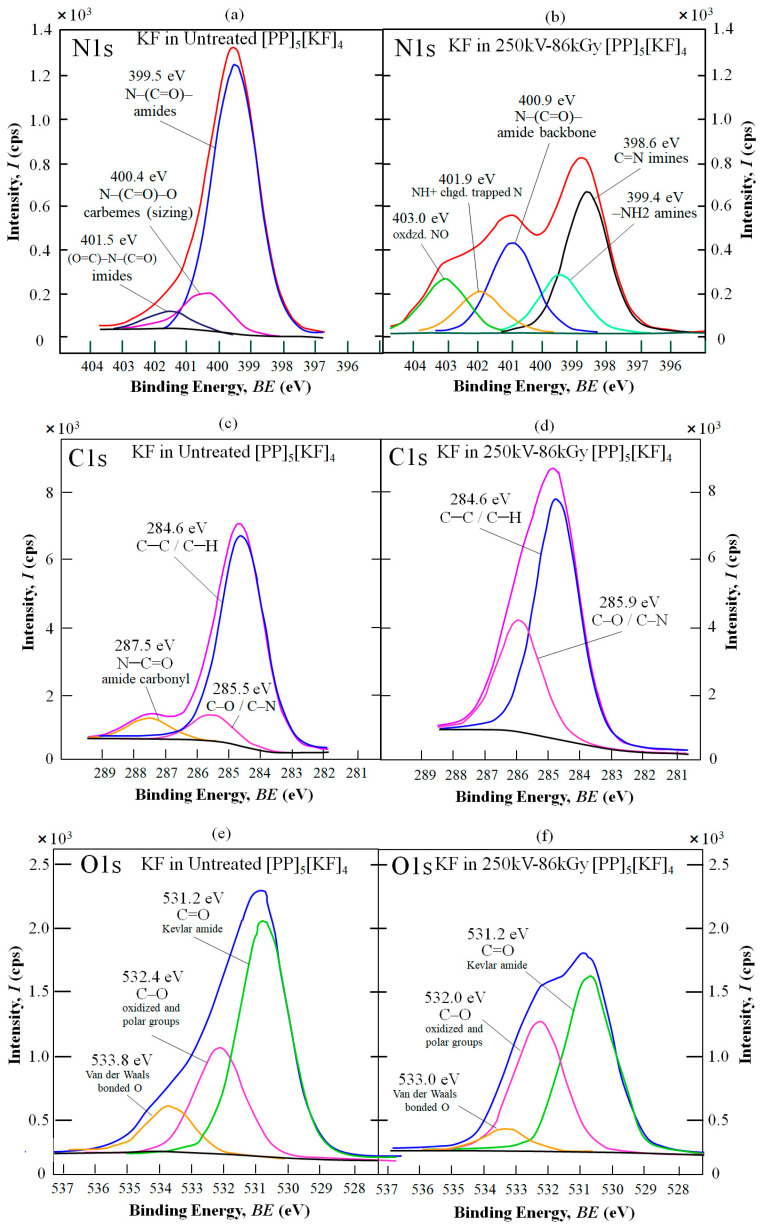
XPS signals of KF in untreated and HLEBI-treated (250 kV–86 kGy) [PP]_5_[KF]_4_ samples in the fracture area. Plots of untreated and treated are (**a**,**b**) N1s, (**c**,**d**) C1s, and (**e**,**f**) O1s, respectively. Envelopes (sum of all component peaks) are in red, purple and blue for the N1s, C1s and O1s spectra, respectively.

To describe the nano-strengthening mechanism, Figure 13a–f show the resulting XPS spectra: N1s (a,b), C1s (c,d), and O1s (e,f), respectively. Peaks are assigned according to the literature [67,75]. These are of the Kevlar fiber surface in the fracture region of untreated and 250-86 kGy HLEBI-treated [PP]_5_[KF]_4_ samples. Overall, they indicate that the 250 kV–86 kGy HLEBI enhances adhesion at the KF/PP interface by creating strong bonds at the KF/PP interface, increasing polar groups, reducing weak Van der Waals forces, and generating a strong active nitrogen-containing interphase. These act to reduce fiber pullout to increase the impact strength of the [PP]_5_[KF]_4_ composite system. The three spectra taken are described below.

**N1s SPECTRUM**

Figure 13a of the N1s spectrum of KF from the untreated sample shows a dominant peak at 399.5 eV binding energy (BE) with 82.7% area, which is characteristic of the Kevlar backbone N–(C=O)– [67]. Since XPS detects from the top 5 to 10 nm from the surface, this indicates a clean fracture between KF and PP in the form of fiber pullout due to poor KF/PP adhesion. A carbeme N–(C=O)–O peak at 400.4 eV characteristic of KF sizing and an imide (C=O)–N–(C=O) peak at 401.5 eV corresponding with the KF are also present.

However, in Figure 13b, showing the HLEBI-treated sample, an XPS scan of the KF shows a wide variety of N species generated as five peaks. First, the dominant peak is shifted to that of strongly bonded imines, –C=N– at 398.6 eV at 36.8%, indicating strong bonds being formed at the KF/PP interface. Secondly, the Kevlar backbone peak N–(C=O)– (400.9 eV) is significantly reduced from 82.7% area of the untreated to 23.8%, indicating increased coverage of the KFs by PP or the N-containing interphase. Thirdly, an amine peak –NH_2_ is present at 399.4 eV, indicating different species of N bonding with PP, assisting in increased KF/PP adhesion. Fourthly, as assigned in other studies [75], the peak at 403.0 eV can be represented as oxidized NO species, the O coming from the 300 ppm O_2_ in the HLEBI chamber. Finally, a peak at 401.9 eV indicates NH+ charged species representing a trapped N charged from the HLEBI, known as DBs which are a type of trapped radicals [75] that can increase nano-compression strength in solids by the repulsive force between DBs. The polar groups along with increased DBs are excellent for increasing the KF surface energy to increase wettability with PP. With the wide variety of N species generated, the present data suggest a strong active nitrogen-containing interphase, as a concentrated interpenetrating network between KF and PP around the KF, acting to increase KF/PP adhesion. This acts as a shock absorber, blunting crack tips during impact to increase the impact strength of the composite.

**C1s SPECTRUM**

Supporting the N1s data, C1s scans in Figure 13c,d show an amide carbonyl group N–C=O peak at 287.5 eV on the KF of the untreated sample (c) that disappears in the HLEBI-treated (d), which is consistent with increased PP adhesion and the SEM photomicrographs in Figure 9. The N–C=O peak could have originated from the KF or its sizing. Nevertheless, it has disappeared, which is evidence that PP or an interphase is covering the KF.

In addition, the C–C/C–H peak area at 284.6 eV is increased from 68.0% to 81.5%, further indicating increased KF/PP adhesion. The C–O/C–N peak area at 285.5 to 285.9 eV is increased from 10.3% to 32.0% by the HLEBI, indicating an increase in polar O groups activating the KF surface and increasing wettability. The O groups arise from the O_2_ in the HLEBI chamber. The N-group increase can also be explained by N_2_ gas in the HLEBI processor and HLEBI generating DBs as lone pair electrons in the PP and KF surfaces in bonding together.

**O1s SPECTRUM**

Likewise, O1s scan data of KF in Figure 13e,f support their N1s and C1s counterparts. Here, three O1s peaks are present. The dominant C=O Kevlar amide peak at 531.2 eV is reduced by the HLEBI indicating increased PP adhesion or N-interphase cover. The increased interphase cover is supported by N1s with its wide variety of N species generated and C1s with disappearance of the N–C=O peak. The enlargement of the ~532 eV peak supports that HLEBI increases oxidized polar groups C-O while the ~533 eV peak characterizes decreasing weak Van der Waals bonded O from the 250 kV–86 kGy HLEBI.

### 4.4. Illustration of Nano-Strengthening Mechanism

**Figure 14:**
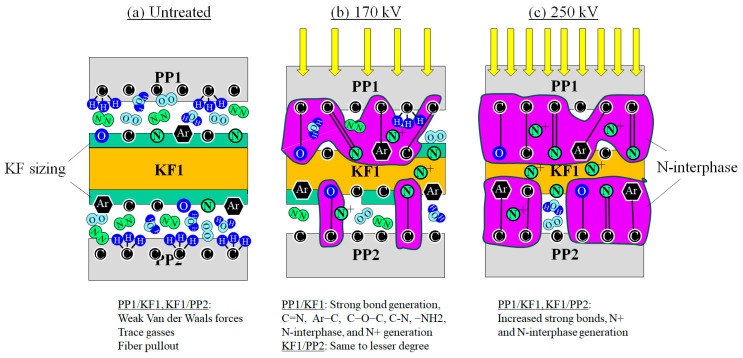
Schematic drawing of PP1/KF1 and KF1/PP2 interfaces near the outside sample surface in KFRTP (**a**) untreated and treated with (**b**) 170 kV and (**c**) 250 kV HLEBI. Purple areas symbolize the N-interphase that overlaps into both KF and PP. ‘Ar’ symbolizes Kevlar aromatic groups. Drawing not to scale. Note HLEBI is applied to both specimen surfaces. Only the outer three plies of one surface are shown here.

Based on the XPS and ESR data, Figure 14 illustrates a simplified nano-strengthening mechanism of the KF/PP interfaces by HLEBI to the outer PP1 surface of the [PP]_5_[KF]_4_ interlayered samples. As shown in Figure 14a, in untreated specimen, nitrogen, oxygen and water (N_2_, O_2_, H_2_O) gas molecules should exist in the KF/PP interfacial spaces due to N_2_ and 300 ppm O_2_ in the HLEBI processor and trace atmospheric gases [76]. The slight attractive force of weak molecular bonding of KF–(N_2_, O_2_, H_2_O)-PP cannot largely be attributed to the KF/PP adhesive force. Mechanical friction with point contacts mainly dominates the resistance to pull out KF from PP, resulting in weak *a*_uc_ (see Figure 5 and Figure 8) for untreated KFRPP.

However, to raise the impact strength, the XPS data indicate that the action of the HLEBI generates an N-containing interphase at the KF/PP interface. In Figure 14b,c it is depicted as purple areas extending into both KF and PP, consisting of strong bonds: imines C=N, and Ar–C, C–O–C, C–N. Figure 14b,c also depict –NH_2_ groups, oxidized NO, and NH+ trapped N as DBs. The chemical species exist in increasingly low density with depth. In the HLEBI-treated specimens, the amount of trace atmospheric gasses H_2_O, N_2_, O_2_ are depicted as decreased compared with the untreated specimens due to the decreased nano-space at the KF/PP interfaces.

To obtain a high increase of 59% at the 250 kV condition, there needs to be an increased density of strong bond generation at the PP1/KF1 and in particular the deeper KF1/PP2 interface, as illustrated in Figure 14b. This is due to the increased HLEBI penetration of *D*_th_ into the KF1 ply, which is closer to the KF1/PP2 interface (see Figure 11) possibly by charge migration. At the deeper KF1/PP2 interface, the 250 kV–86 kGy will exhibit higher strong bond density than that of the 170 kV–129 kGy condition to raise the *a*_uc_. XPS data were not available for 170 kV condition; hence, Figure 13b is constructed as intermediate, having less effect than 250 kV.

On the other hand, Figure 8 shows that the *a*_uc_ decay in [PP]_5_[KF]_4_ irradiated at higher *V*_c_ and doses occurs due to excess DBs weakening the composite structure. Therefore, carefulness is needed to adjust for optimum HLEBI *V*_c_–dose combinations in practical applications.

Although the percent contribution of the KF/PP interface and PP matrix strengthening could not be isolated quantitatively in this paper, the XPS and SEM results support the KF/PP interface strengthening to be dominant. This is evidenced by XPS N1s and C1s peaks indicating an increased coverage of KF by PP, the KF itself not appearing to be chemically modified, and the SEM of the PP matrix remaining on the KF as enhanced adhesion. Secondary contribution is apparently due to DB generation in the PP itself.

## 5. Conclusions

Presently, there is little or no literature found that evaluates the effect of electron beams on *para*-aramid (Kevlar^®^) fiber polymer (KFRP) composites. Therefore, we investigated the effect of homogeneous low-voltage electron beam irradiation (HLEBI) on KFR-polypropylene (KFRPP) interlayered samples. The results showed when a cathode voltage–dose combination of 250 kV–86 kGy was applied to both sides of the finished samples, the Charpy impact value was raised 59% compared to the untreated sample. The 250 kV–86 kGy combination raised the impact value more than that at 170 kV–129 kGy (15%) due to the increased penetration depth from the higher cathode voltage. Scanning electron microscopy (SEM) revealed that the HLEBI increases KF/PP adhesion, reducing fiber pullout. On a nanoscale level, electron spin resonance (ESR) analysis showed the HLEBI generates dangling bonds (DBs) in both KF and PP; i.e., lone pair electrons that are electrical repulsion sites creating nano-internal compressive stress to strengthen materials, crosslinking, and activating surfaces for strong bonding, particularly of hard-to-adhere components. Moreover, the X-ray photoelectron spectroscopy (XPS) N1s spectrum of Kevlar fiber from the fracture region showed a shift in the dominant peak of N–(C=O)– in the untreated sample to that of strongly-bonded imines, –C=N– in the 250 kV–86 kGy HLEBI sample, indicating strong bonds formed at the KF/PP interface. Together N1s, C1s and O1s spectra indicated increased polar groups, reduced weak Van der Waals forces, and the generation of a strong active nitrogen-containing interphase, acting to reduce fiber pullout to increase the impact strength of the [PP]_5_[KF]_4_ composite system.

This paper can help increase the important property of impact strength of KFRPP aerospace and automotive parts, protective articles in industry, structural components, and sports equipment. It has the advantage of using a lightweight material containing recyclable thermoplastic polypropylene and HLEBI treatment that uses no chemical treatments; in addition, it does not produce waste and takes only seconds. Future investigations should include tensile, compression, interlaminar shear, fatigue, aging, and environmental factors such as ultraviolet or cosmic rays, temperature and humidity.

## Figures and Tables

**Table 1:** Test matrix for HLEBI treatment of Kevlar fiber [PP]_5_[KF]_4_ specimens. Underlined, bold values indicate highest impact value attained.

*V*_c_ (kV)	Dose (kGy)	Number of Specimens/Data Set
Untreated	0	(Untreated) 17
170 kV	43.2, 86, **129**, 172, 229, 258, 301	(170 kV–129 kGy) 14 (all others, 3)
250 kV	43.2, **86**, 129, 172, 229, 258, 301	(250 kV–86 kGy) 14 (all others, 3)

**Table 2:** Estimated *V*_x(PP1)_ (kV) at PP1 ply outer surface of sample from *V*_c_ setting and dropped potentials (Δ*V*_Ti_ and Δ*V*_N2_) across the Ti window and N_2_ atmosphere of the HLEBI processor.

Cathode Potential (*V*_c_: kV)	Dropped Potential		Surface Electrical Potential at PC1 Surface (*V*_x(PP1)_: kV) (*x* = 170 or 250)	
	in Ti-Window (Δ*V*_Ti_: kV)	in N_2_ Gas Atmosphere (Δ*V*_N2_: kV)		Calculated Penetration Depth into Outer PP1 ply (*D*_th_: μm)
170	22.2	15.1	132.7	256
250	17.2	11.2	221.7	602

**Table 3:** Average *a*_uc_ and standard deviations for the data sets in Figure 5.

	*a*_uc_ (Avg.) (kJm^–2^)	*a*_uc_ at *P*_f_ = 0.50 (kJm^–2^)	Weibull Modulus, *n*
Untreated	74.3 (8.4)	72.5	9.76
170 kV–129 kGy	83.6 (10.6)	83.3	8.71
250 kV–86 kGy	113.0 (10.2)	115.6	11.67

**Table 4:** Thicknesses and densities along with *V*_x_ and *D*_th_ values at each *V*_c_ setting for each successive layer into specimen thickness according to Equations (11) and (12).

	Thickness	Density	Voltage at Top Surface of Layer (*V*_X_: kV)/ Penetration Depth from Top Surfaces (*D*_th_: μm)			
Material	*T* (μm)	*ρ* (kg/m^3^)	170 kV		250 kV	
(ply)			*V*_170_	*D*_th_	*V*_250_	*D*_th_
Ti window	10	4540	170	77	250	146
N_2_	25,000	1.13	148	243,932	233	520,199
PP1	200	900	133	256	222	602
KF1	200	1440	29	**13**	148	**192**
PP2	200	900	0	0	0	0
KF2	200	1440	0	0	0	0

## Data Availability

The original contributions presented in this study are included in the article. Further inquiries can be directed to the corresponding author.

## Abbreviations

The following abbreviations are used in this manuscript:

KFRP	Kevlar Fiber-Reinforced Polymer
KFRPP	Kevlar Fiber-Reinforced Polypropylene
HLEBI	Homogeneous Low-Potential Electron Beam Irradiation
DB	Dangling Bond
para-AF	para-Aramid Fiber
